# [Expression of Concern] Lentivirus-mediated RNA interference targeting UbcH10 reduces cell growth and invasion of human osteosarcoma cells via inhibition of Ki-67 and matrix metalloproteinases 

**DOI:** 10.3892/ol.2025.15260

**Published:** 2025-09-08

**Authors:** Shan-Tao Wang, Dan-Zhi Li, Jian-Min Li, Jun Fang, Hua-Zhuang Li, Pei-Jian Tong, Fu-Cun Liu

Oncol Lett 9: 2171–2176, 2015; DOI: 10.3892/ol.2015.3023

Following the publication of this paper, it was drawn to the Editor's attention by a concerned reader that, regarding the scratch-wound assay experiments shown in Fig. 3C on p. 2175, the 24 h SaOS2 and U2OS panels on the left appeared to be overlapping, suggesting that these data had been assembled incorrectly in this figure (in fact, a subsequent analysis of the data in this paper revealed that there was a second pair of overlapping data panels in this figure).

The authors were contacted by the Editorial Office to offer an explanation for these apparent anomalies in the presentation of the data in this paper; however, up to this time, no response from them has been forthcoming. Owing to the fact that the Editorial Office has been made aware of potential issues surrounding the scientific integrity of this paper, we are issuing an Expression of Concern to notify readers of this potential problem while the Editorial Office continues to investigate this matter further.

